# Supplementary material for: Machine Learning Accelerates Crystallization for Structure Determination
Source: Angew Chem Int Ed Engl. 2026 May 4;65(25):e1218503. doi: 10.1002/anie.1218503 (PMC13266958; doi:10.1002/anie.1218503)

checkCIF/PLATON report

Structure factors have been supplied for datablock(s) lcz087

THIS REPORT IS FOR GUIDANCE ONLY. IF USED AS PART OF A REVIEW PROCEDURE FOR PUBLICATION, IT SHOULD NOT REPLACE THE EXPERTISE OF AN EXPERIENCED CRYSTALLOGRAPHIC REFEREE.

No syntax errors found. CIF dictionary Interpreting this report

Datablock: lcz087

Bond precision:	C-C = 0.0105 A		Wavelength=1.54184
Cell:	a=21.9920(2)	b=12.7594(1)	c=24.7580(3)
	alpha=90	beta=103.839(1)	gamma=90
Temperature:	100 K		

	Calculated	Reported
Volume	6745.55(12)	6745.54(12)
Space group	P 21/c	P 1 21/c 1
Hall group	-P 2ybc	-P 2ybc
Moiety formula	C27 H12 Ag3 F18 N7 S, C15 H3 Ag3 F18 N6, C12 H9 N S	C15 H3 Ag3 F18 N6, C12 H9 N S
Sum formula	C54 H24 Ag6 F36 N14 S2	C27 H12 Ag3 F18 N7 S
Mr	2264.21	1132.11
Dx,g cm-3	2.230	2.230
Z	4	8
Mu (mm-1)	15.676	15.676
F000	4336.0	4336.0
F000’	4358.91	
h,k,lmax	27,16,31	27,16,30
Nref	14483	13687
Tmin,Tmax	0.109,0.043	0.317,1.000
Tmin’	0.017	

Correction method= # Reported T Limits: Tmin=0.317 Tmax=1.000
AbsCorr = MULTI-SCAN

Data completeness= 0.945 Theta(max)= 78.402

R(reflections)= 0.0495(11862) wR2(reflections)= 0.1435(13687)

S = 1.049 Npar= 1009

The following ALERTS were generated. Each ALERT has the format
test-name_ALERT_alert-type_alert-level.
Click on the hyperlinks for more details of the test.

Alert level C

```
PLAT041_ALERT_1_C Calc. and Reported SumFormula    Strings  Differ     Please Check
              Calc: C54 H24 Ag6 F36 N14 S2
              Rep.: C27 H12 Ag3 F18 N7 S
PLAT042_ALERT_1_C Calc. and Reported MoietyFormula Strings  Differ     Please Check
              Calc: C27 H12 Ag3 F18 N7 S, C15 H3 Ag3 F18 N6, C12 H
                    9 N S
              Rep.: C15 H3 Ag3 F18 N6, C12 H9 N S
PLAT214_ALERT_2_C Atom F01V      (Anion/Solvent) ADP max/min Ratio        4.2 prolat
PLAT221_ALERT_2_C Solv./Anion  Resd 2  F   Ueq(max)/Ueq(min) Range        4.3 Ratio
PLAT250_ALERT_2_C Large U3/U1 Ratio for <U(i,j)> Tensor(Resd    2)        2.7 Note
PLAT342_ALERT_3_C Low Bond Precision on  C-C Bonds ..............    0.01046 Ang.
PLAT420_ALERT_2_C D-H Bond Without Acceptor  N01H     --H01H     .     Please Check
PLAT906_ALERT_3_C Large K Value in the Analysis of Variance ......      3.872 Check
PLAT911_ALERT_3_C Missing FCF Refl Between Thmin & STh/L=    0.600        136 Report
                 1  1  0,   2  0  0,   6  7  0,  -7  7  1,  -2  1  1,   2  1  1,
                 6 12  1,   7 11  1,   8  6  1,   9  6  1,  10  6  1,  -2  1  2,
                 3  0  2,   4  0  2,   6 12  2,   7  6  2,   7 12  2,  18  9  2,
                20  9  2,  21  8  2,   5 14  3,   6 12  3,   6 14  3,   7 14  3,
                -2  0  4,   0  0  4,   4 14  4,   2 14  5,  10  3  5,   8  4  7,
                   (  106 More Missing: see the .ckf listing file)
PLAT977_ALERT_2_C Check Negative Difference Density on H01H      .      -0.34 eA-3
PLAT977_ALERT_2_C Check Negative Difference Density on H01J      .      -0.65 eA-3
PLAT977_ALERT_2_C Check Negative Difference Density on H02O      .      -0.32 eA-3
PLAT977_ALERT_2_C Check Negative Difference Density on H02X      .      -0.34 eA-3
PLAT977_ALERT_2_C Check Negative Difference Density on H032      .      -0.32 eA-3
```

Alert level G

```
PLAT007_ALERT_5_G Number of Unrefined Donor-H Atoms .............          2 Report
              H01J  H01H
PLAT045_ALERT_1_G Calculated and Reported Z Differ by a Factor ...      0.500 Check
PLAT083_ALERT_2_G SHELXL Second Parameter in WGHT  Unusually Large      35.68 Why ?
PLAT142_ALERT_4_G s.u. on b - Axis Small or Missing .............    0.00010 Ang.
PLAT242_ALERT_2_G Low    'MainMol' Ueq as Compared to Neighbors of       C027 Check
PLAT242_ALERT_2_G Low    'MainMol' Ueq as Compared to Neighbors of       C02B Check
PLAT242_ALERT_2_G Low    'MainMol' Ueq as Compared to Neighbors of       C02K Check
PLAT242_ALERT_2_G Low    'MainMol' Ueq as Compared to Neighbors of       C02S Check
PLAT242_ALERT_2_G Low    'MainMol' Ueq as Compared to Neighbors of       C02T Check
PLAT242_ALERT_2_G Low    'MainMol' Ueq as Compared to Neighbors of       C02W Check
PLAT242_ALERT_2_G Low    'MainMol' Ueq as Compared to Neighbors of       C01U Check
PLAT242_ALERT_2_G Low    'MainMol' Ueq as Compared to Neighbors of       C022 Check
PLAT242_ALERT_2_G Low    'MainMol' Ueq as Compared to Neighbors of       C023 Check
```

```
PLAT242_ALERT_2_G Low    'MainMol' Ueq as Compared to Neighbors of       C02I Check
PLAT242_ALERT_2_G Low    'MainMol' Ueq as Compared to Neighbors of       C02J Check
PLAT242_ALERT_2_G Low    'MainMol' Ueq as Compared to Neighbors of       C034 Check
PLAT434_ALERT_2_G Short Inter HL..HL Contact F00D     ..F00P     .       2.81 Ang.
                                         -x,-1/2+y,1/2-z  =      2_545 Check
PLAT434_ALERT_2_G Short Inter HL..HL Contact F00T     ..F01L     .       2.76 Ang.
                                                x,-1+y,z  =      1_545 Check
PLAT720_ALERT_4_G Number of Unusual/Non-Standard Labels .........         136 Note
              Ag01    Ag02    Ag03    Ag04    Ag05    Ag06    S007    S008
              F009    F00A    F00B    F00C    F00D    F00E    F00F    F00G
              F00H    F00I    F00J    N00K    F00L    F00M    N00N    F00O
              F00P    N00Q    F00R    N00S    F00T    F00U    N00V    N00W
              N00X    F00Y    N00Z    F010    F011    F012    F013    N014
              F015    N016    F017    F018    F019    F01A    F01B    N01C
              F01D    F01E    N01F    F01G    N01H    H01H    F01I    N01J
              H01J    C01K    H01K    F01L    C01M    C01N    C01O    C01P
              C01Q    C01R    C01S    C01T    C01U    F01V    C01W    C01X
              H01X    C01Y    H01Y    C01Z    H01Z    C020    H020    C021
              H021    C022    C023    C024    C025    C026    C027    C028
              C029    H029    C02A    C02B    C02C    H02C    C02D    C02E
              C02F    C02G    H02G    C02H    C02I    C02J    C02K    C02L
              H02L    C02M    C02N    H02N    C02O    H02O    C02P    H02P
              C02Q    C02R    H02R    C02S    C02T    C02U    H02U    C02V
              H02V    C02W    C02X    H02X    C02Y    H02Y    C02Z    H02Z
              C030    H030    C031    H031    C032    H032    F033    C034
PLAT883_ALERT_1_G Absent Datum for _atom_sites_solution_primary ..     Please Do !
PLAT910_ALERT_3_G Missing FCF Reflection(s) Below Theta(Min)[Deg]=       3.68 Note
                1  0  0,
PLAT912_ALERT_4_G Missing # of FCF Reflections Above STh/L=  0.600        645 Note
PLAT933_ALERT_2_G Number of HKL-OMIT Records in Embedded .res File         10 Note
              -10  3 28, -11  6 27, -12  2 26, -14  3 24,  -9  2 28, -10  5 28,
              -12  0 24, -12  3 26, -11  3 27, -12  0 22,
PLAT941_ALERT_3_G Average HKL Measurement Multiplicity ..........         2.3 Low
PLAT969_ALERT_5_G The 'Henn et al.' R-Factor-gap value ..........      2.612 Note
              Predicted wR2: Based on SigI**2  5.49 or SHELX Weight 13.67
PLAT978_ALERT_2_G Number C-C Bonds with Positive Residual Density.          0 Info
```

```
   0 ALERT level A = Most likely a serious problem - resolve or explain
   0 ALERT level B = A potentially serious problem, consider carefully
  14 ALERT level C = Check. Ensure it is not caused by an omission or oversight
  26 ALERT level G = General information/check it is not something unexpected

   4 ALERT type 1 CIF construction/syntax error, inconsistent or missing data
  26 ALERT type 2 Indicator that the structure model may be wrong or deficient
   5 ALERT type 3 Indicator that the structure quality may be low
   3 ALERT type 4 Improvement, methodology, query or suggestion
   2 ALERT type 5 Informative message, check
```

Validation response form

Please find below a validation response form (VRF) that can be filled in and pasted into your CIF.

```
# start Validation Reply Form
_vrf_PLAT041_lcz087
;
PROBLEM: Calc. and Reported SumFormula    Strings  Differ     Please Check
RESPONSE: ...
;
_vrf_PLAT042_lcz087
;
PROBLEM: Calc. and Reported MoietyFormula Strings  Differ     Please Check
RESPONSE: ...
;
_vrf_PLAT214_lcz087
;
PROBLEM: Atom F01V      (Anion/Solvent) ADP max/min Ratio        4.2 prolat
RESPONSE: ...
;
_vrf_PLAT221_lcz087
;
PROBLEM: Solv./Anion  Resd 2  F   Ueq(max)/Ueq(min) Range        4.3 Ratio
RESPONSE: ...
;
_vrf_PLAT250_lcz087
;
PROBLEM: Large U3/U1 Ratio for <U(i,j)> Tensor(Resd    2)        2.7 Note
RESPONSE: ...
;
_vrf_PLAT342_lcz087
;
PROBLEM: Low Bond Precision on  C-C Bonds ..............    0.01046 Ang.
RESPONSE: ...
;
_vrf_PLAT420_lcz087
;
PROBLEM: D-H Bond Without Acceptor  N01H     --H01H     .     Please Check
RESPONSE: ...
;
_vrf_PLAT906_lcz087
;
PROBLEM: Large K Value in the Analysis of Variance ......      3.872 Check
RESPONSE: ...
;
_vrf_PLAT911_lcz087
;
PROBLEM: Missing FCF Refl Between Thmin & STh/L=    0.600        136 Report
RESPONSE: ...
;
_vrf_PLAT977_lcz087
;
PROBLEM: Check Negative Difference Density on H01H      .      -0.34 eA-3
RESPONSE: ...
;
# end Validation Reply Form
```

It is advisable to attempt to resolve as many as possible of the alerts in all categories. Often the minor alerts point to easily fixed oversights, errors and omissions in your CIF or refinement strategy, so attention to these fine details can be worthwhile. It is up to the individual to critically assess their own results and, if necessary, seek expert advice.

PLATON version of 17/09/2025; check.def file version of 17/09/2025

---

duplicate check

No duplication found

Datablock lcz087 - ellipsoid plot

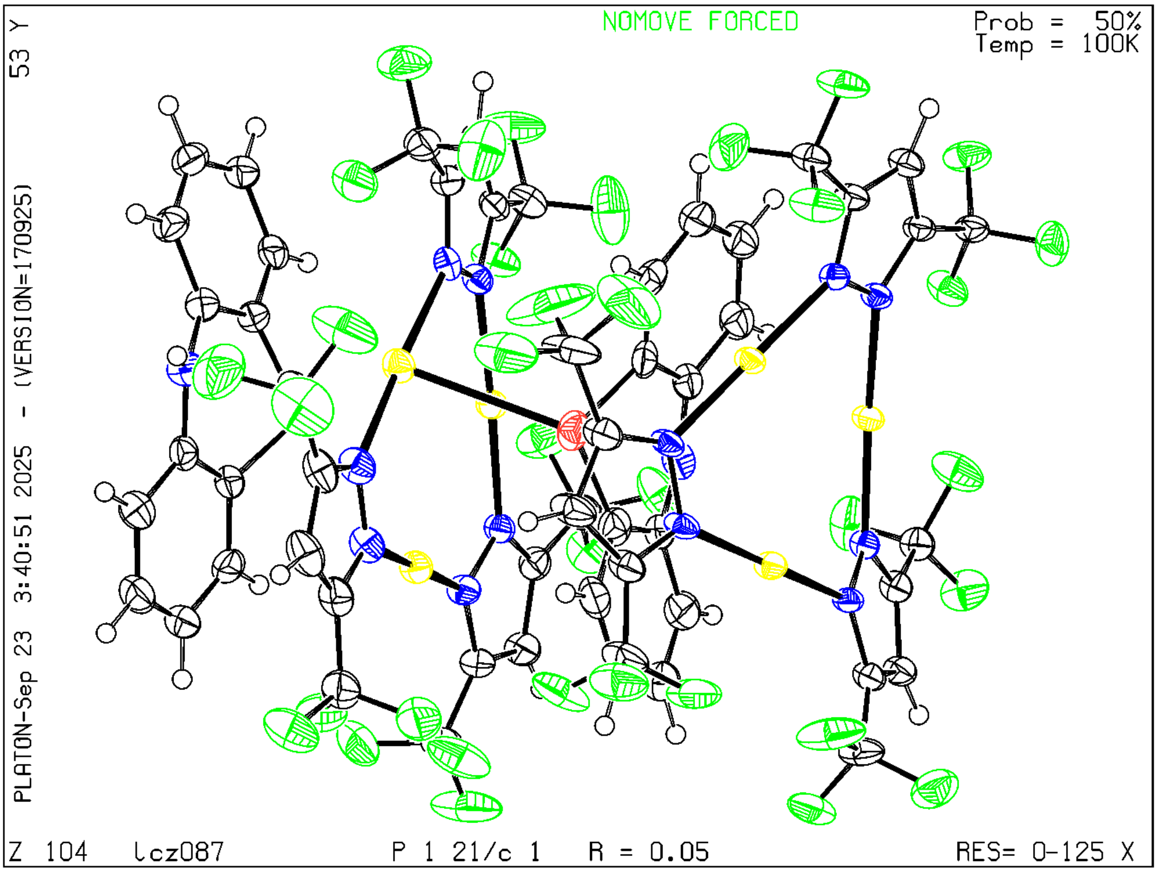

Supplement: Supplementary file 2 — Supporting File 2: anie72462‐sup‐0002‐DataFile.zip. [file ANIE-65-e1218503-s002.zip › supplementary_materials_crystallographic_data submit.zip!/cif_and_checkcif(1-114)/Ag3Pz3_87/Ag3Pz3_87_checkcif.pdf]
